# An *in vitro* cell irradiation protocol for testing photopharmaceuticals and the effect of blue, green, and red light on human cancer cell lines†
†Electronic supplementary information (ESI) available. See DOI: 10.1039/c5pp00424a



**DOI:** 10.1039/c5pp00424a

**Published:** 2016-03-31

**Authors:** S. L. Hopkins, B. Siewert, S. H. C. Askes, P. Veldhuizen, R. Zwier, Michal Heger, Sylvestre Bonnet

**Affiliations:** a Leiden Institute of Chemistry , Leiden University , Einsteinweg 55 , 2300RA Leiden , The Netherlands . Email: bonnet@chem.leidenuniv.nl; b Leiden Institute of Physics , Leiden University , Niels Bohrweg 2 , 2333CA Leiden , The Netherlands; c Department of Experimental Surgery , Academic Medical Center , University of Amsterdam , Meibergdreef 9 , 1105 AZ Amsterdam , The Netherlands

## Abstract

Traditionally, ultraviolet light (100–400 nm) is considered an exogenous carcinogen while visible light (400–780 nm) is deemed harmless. In this work, a LED irradiation system for *in vitro* photocytotoxicity testing is described. The LED irradiation system was developed for testing photopharmaceutical drugs, but was used here to determine the basal level response of human cancer cell lines to visible light of different wavelengths, without any photo(chemo)therapeutic. The effects of blue (455 nm, 10.5 mW cm^–2^), green (520 nm, 20.9 mW cm^–2^), and red light (630 nm, 34.4 mW cm^–2^) irradiation was measured for A375 (human malignant melanoma), A431 (human epidermoid carcinoma), A549 (human lung carcinoma), MCF7 (human mammary gland adenocarcinoma), MDA-MB-231 (human mammary gland adenocarcinoma), and U-87 MG (human glioblastoma-grade IV) cell lines. In response to a blue light dose of 19 J cm^–2^, three cell lines exhibited a minimal (20%, MDA-MB-231) to moderate (30%, A549 and 60%, A375) reduction in cell viability, compared to dark controls. The other cell lines were not affected. Effective blue light doses that produce a therapeutic response in 50% of the cell population (ED_50_) compared to dark conditions were found to be 10.9 and 30.5 J cm^–2^ for A375 and A549 cells, respectively. No adverse effects were observed in any of the six cell lines irradiated with a 19 J cm^–2^ dose of 520 nm (green) or 630 nm (red) light. The results demonstrate that blue light irradiation can have an effect on the viability of certain human cancer cell types and controls should be used in photopharmaceutical testing, which uses high-energy (blue or violet) visible light activation.

## Abstract 2

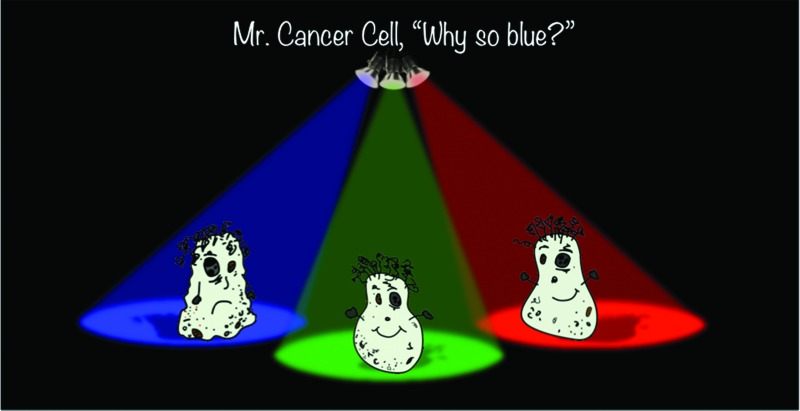
A LED-based cell irradiation system was built that can irradiate a 96-well plate with monochromatic light at controlled temperature and with a built-in dark control. This system was used to study the response of six human cancer cell lines to blue, green, and red light.

## Introduction

For several decades solar UVA (ultraviolet-A, 320–400 nm) and UVB (ultraviolet-B, 290–320 nm) irradiation have been considered environmental carcinogens that contribute to the development of skin cancer. Ultraviolet light interacts with endogenous photosensitizers stimulating reactive oxygen species (ROS) generation, free radical accumulation, and oxidative stress. When persistent, these processes culminate in irreversible cascades of mutagenic processes such as DNA strand breaks, pyrimidine dimerization, lipid peroxidation, protein damage, and cellular stress responses.^1–5^ In contrast, visible light (400–780 nm), which represents more than 50% of the solar spectrum,^6^ is generally considered non-toxic to cells.

In cancer photochemotherapy, which includes photodynamic therapy (PDT)^7–13^ and photoactivated chemotherapy (PACT),^14–22^ intense visible light is used to induce a drug response in cancer cells, whereas a minimal chemotherapeutic response occurs in the dark. Typically, light-induced drug activation occurs *via* generation of lethal oxidative stress (in PDT), or release of a caged compound that becomes cytotoxic (in PACT). Several considerations must be taken into account when testing photopharmaceuticals *in vitro*, such as cell type, light sources, cell environment, and cell counting assays. In addition, several of the endogenous photosensitizers (flavins, porphyrins, bilirubin, and melanin) that mediate oxidative damage by UVA irradiation, also strongly absorb high-energy visible light (HEVL, 400–500 nm).^23–25^ Consequently, cytotoxic effects of visible light may occur even in the absence of any photopharmacologically active compound.

The goal of this study was two-fold. Part one aims to provide a LED-based cell irradiation device and a protocol for *in vitro* testing of photopharmacologically active compounds with full characterization of the irradiation system. This system can irradiate cells in standard 96-well plates at controlled temperature, with controlled light intensity at three different wavelengths (455, 520, 630 nm) under the same controlled dark conditions. A survey of the literature revealed that experimental conditions for *in vitro* cell testing under light irradiation vary drastically, which makes comparison difficult. Meanwhile, poorly described irradiation setups make it nearly impossible to reproduce many studies. We address these issues by thoroughly describing our cell irradiation device, as well as the protocol. In the second part of the paper, we use this setup to determine the cytotoxicity of blue, green, and red light towards six human cancer cell lines commonly used for *in vitro* testing of light-activated pharmacological compounds (skin, breast, lung, and brain).

## Results

### Building a visible light irradiation device for *in vitro* cell testing

**Fig. 1:**
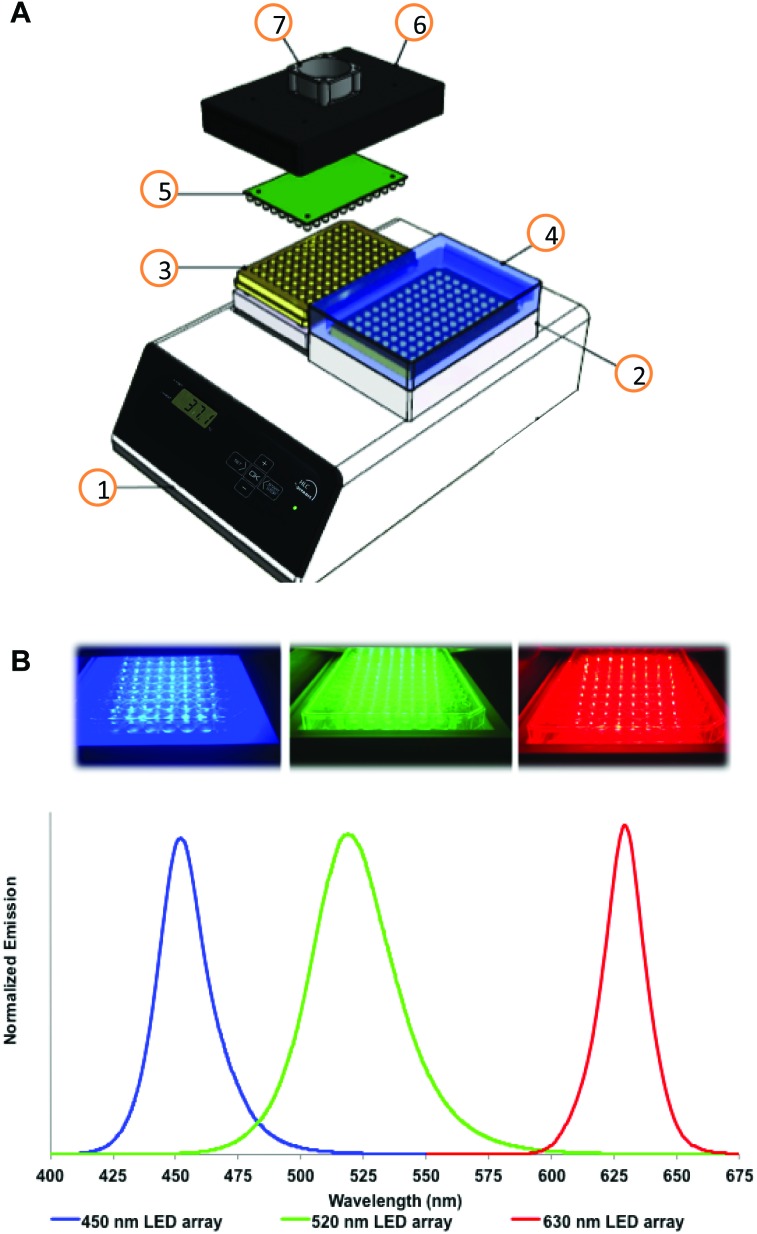
Experimental setup of the *in vitro* cell irradiation system (A), with photographs and emission spectra for blue, green, and red LED arrays (B). The cell irradiation system consists of a Ditabis thermostat (1), two flat-bottomed 96-well plate thermoblocks (2), two 96-well plates (3), cover for the dark control sample shown as transparent for clarity (4), a 96-LED array mounted on a printed circuit board (5), cover for a 96-LED array (6), and a fan for cooling the LED array (7).

*In vitro* testing of photopharmaceutical compounds relies on performing reproducible cytotoxicity tests under controlled light irradiation. Thus, a cell irradiation device compatible with standard 96-well plates was developed. More specifically, the LED irradiation setup allows for simultaneously running “dark” and “irradiated” experiments under identical conditions (Fig. 1). A thermostat fitted with flat-bottom microtiter plate thermoblocks was used to maintain a constant and equal temperature in both plates while one plate is irradiated. Temperature control was included in the design as many photochemical reactions are temperature-dependent. In addition, when simulating *in vivo* irradiation, an *in vitro* setup should be able to maintain a temperature of ∼37 °C rather than room temperature. Though multiple LED arrays of any wavelength can be imagined, three LED arrays are thoroughly described here allowing for irradiating cells with blue, green, or red light. A full technical description of the irradiation device and LED arrays is provided in the ESI.†

Several parameters of the cell irradiation system were evaluated and are reported in Table 1, including the actual wavelength and half-bandwidth of the LEDs used, the average light power density obtained at the bottom of each well of a 96-well plate, and the thermal stability of the cell-growing medium present in each well (200 μM) under dark and irradiated conditions (see the ESI† for full details of the measurements). For blue and green light, power density was measured using both chemical actinometry and a power meter coupled to an integrating sphere. For blue light, the power density was identical for the two methods, validating the power meter measurement. Chemical actinometry revealed that light distribution was homogeneous within the central 60 wells (Fig. S6†). For green light, some discrepancy (∼20%) was found between the two measurement methods, which was attributed to poor absorption of the ferrioxalate actinometer at 520 nm. For red light we were unable to find a suitable chemical actinometer compatible with aqueous solutions, so only the physical measurement is reported. In all further experiments light doses (Table 2) were calculated using the power density physically measured with the integration sphere. Overall, the 96-well irradiation system provided a consistent blue, green, or red light output over the central 60 wells, while the outer 36 wells were not used to avoid border effects. The temperature in the dark and irradiated wells was comparable within ±1 °C. This setup can be used for the reproducible and convenient testing of light-activated compounds *in vitro*. As an example, two known, soluble, and affordable photodynamic therapy dyes, *i.e.*, rose bengal and methylene blue, were tested with this setup against A375, A431, A549, MCF7, MDA-MB-231, U-87 MG human cancer cell lines (see the ESI†). A summary of the effective concentration values (EC_50_) measured in the dark and under a blue, green, or red light dose of 6 J cm^–2^ is shown in Fig. S8,† with typical dose–response curves in Fig. S9.†

**Table 1:** LED array characterization including emission maximum (nm), power densities at the bottom of each well measured using an integrating sphere or chemical actinometry (mW cm^–2^), and average temperature (°C) of well D6 during irradiation with the blue, green, or red LED arrays^*a*^

Wavelength ± FWHM^*b*^ (nm)	Power density^*c*^ (mW cm^–2^)		Temperature^*e*^ (°C)
	Integrating sphere	Chemical actinometry ^*d*^	
Dark control	—	—	37 ± 1
454 ± 11	10.5 ± 0.7	10.2 ± 0.9	36 ± 1
520 ± 20	20.9 ± 1.6	16.6 ± 0.1	35 ± 1
630 ± 9	34.4 ± 1.7	n.a.	37 ± 1

^*a*^See the ESI for further information.

^*b*^Wavelength was measured using an integrating sphere, FWHM = full width at half maximum.

^*c*^Measured at a set voltage of 28.9, 27.9, and 20.7 V for the blue, green, and red LED arrays, respectively.

^*d*^Average of 3 independent experiments.

^*e*^Measured in the dark or under irradiation over 45 min.

**Table 2:** Light doses (J cm^–2^) *vs.* irradiation time calculated from power densities measured using the integrating sphere for the blue, green, and red LED arrays (Table 1)

Times for blue & green LED array	455 nm light dose (J cm^–2^)	520 nm light dose (J cm^–2^)	Times for red LED array	630 nm light dose (J cm^–2^)
5 min	3 ± 0.2	6 ± 0.5	3 min	6 ± 0.3
10 min	6 ± 0.4	13 ± 1.0	6 min	12 ± 0.6
15 min	9 ± 0.6	19 ± 1.0	9 min	19 ± 0.9
30 min	19 ± 1.0	38 ± 3.0	18 min	37 ± 1.9

### Light cytotoxicity and cell growth curves

**Fig. 2:**
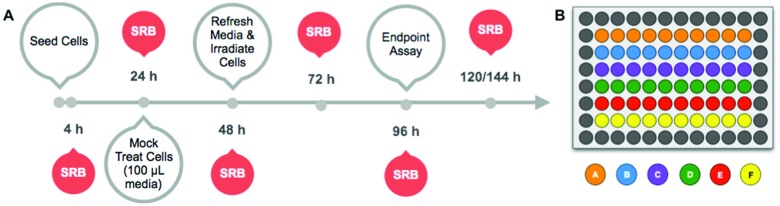
Timeline for optimized cytotoxicity experiments (A) and a 96-well plate setup for cytotoxicity experiments (B). The timeline includes the general experimental setup (grey outlined) and the time points at which the sulforhodamine B (SRB) assay was performed for plotting the growth curve (filled). In the 96-well plate setup, each letter (A–F) corresponds to a cancer cell line; placement was varied to eliminate plating bias. Each plate shows ten technical replicates (*n*_t_ = 10).

**Fig. 3:**
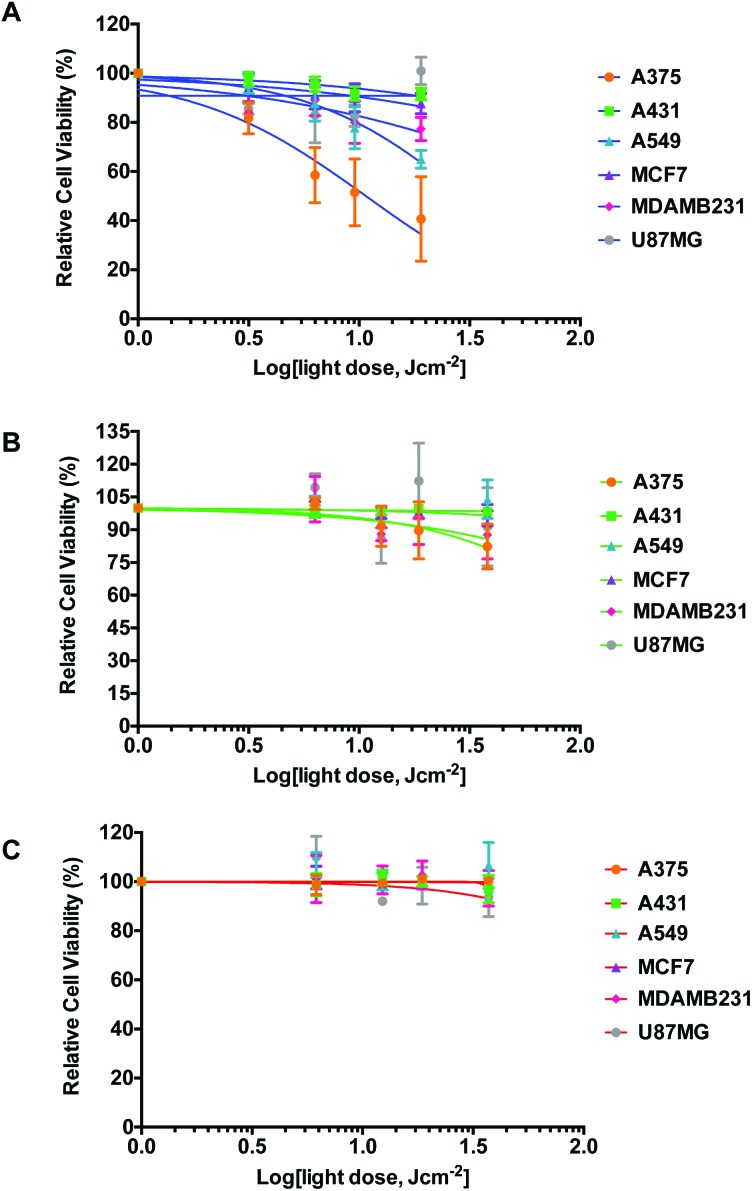
Light dose–response curves for six cancer cell lines following irradiation by the 455 nm (A), 520 nm (B), or 630 nm (C) LED array. Response was calculated as irradiated cell viability divided by dark cell viability. Conditions: cells seeded at *t* = 0 and incubated under standard culturing conditions. Opti-MEM complete without phenol red was added at 24 h and cells were incubated for an additional 24 h (mock treatment). Media were refreshed and cells were irradiated at varied dosages using one of the three 96-well LED arrays; plates were then placed back in the incubator. SRB assay performed at 96 h. Each experiment consisted of ten technical replicates (*n*_t_ = 10), which was repeated three times (*n*_b_ = 3). The dose–response curves (lines) were fitted using a two-parameter Hill-slope analysis.

The light dose–response and the growth curves of cancer cells (A375, A431, A549, MCF7, MDA-MB-231, and U-87 MG) in the absence of any compound were investigated at 455 nm, 520 nm, and 630 nm, and compared to dark controls. The protocol is outlined in Fig. 2. Briefly, the cells were seeded at *t* = 0, incubated in the dark for 24 h, and mock-treated with medium for 24 h. The medium was then changed (*t* = 48 h) and the cells were irradiated with blue, green, or red light. Finally, the cells were further incubated until *t* = 96 h, fixed, and the cell viabilities were evaluated using the SRB assay. Cell irradiation times of 5, 10, 15, or 30 min were used for 450 and 520 nm, and of 3, 6, 9, or 18 min for 630 nm. The light response was plotted as relative cell viability in irradiated plates compared to non-irradiated cells, as a function of log(light dose in J cm^–2^). Each well was considered as a technical replicate; all ten technical replicates (*n*_t_ = 10) from each plate were averaged to form one mean for a single biological replicate. Three biological replicates (*n*_b_ = 3) were completed to determine whether variations in biological processes between passage numbers strongly impacted the effect of light irradiation. The light dose–response curves for the three wavelengths are plotted in Fig. 3. Irradiation by blue light (Fig. 3A) resulted in an unexpected, but substantially reduced cell growth for two cell lines (A375 and A549), while one cell line (MDA-MB-231) displayed minimal effects, and the three remaining cell lines (A431, MCF7, and U-87 MG) displayed negligible differences in cell population between the irradiated and dark plates. For A375 and A549 cells, the blue light dose that reduced cell growth by 50% compared to dark conditions (ED_50_) was calculated using a nonlinear regression fit with a variable Hill-slope and is reported with their ±95% confidence intervals (CI) in Table 3. The ED_50_ values were 10.9 ± 3.0 and 30.5 ± 12.0 J cm^–2^ for the A375 and A549 cell lines, respectively. For MDA-MB-231 and for the cell lines that exhibited no impact of blue light the ED_50_ values could not be calculated without large errors and are not reported. At 520 nm, the irradiation dose–response curves indicated minimal effects of green light on A375 and MDA-MB-231 cells (Fig. 3B) with large errors associated with the mean ED_50_ values (140 and 350 J cm^–2^, respectively). Green light had no effects at all on the other four cell lines. The dose–response curves at 630 nm showed no effects on any of the cell lines investigated (Fig. 3C). Micrographs of irradiated and non-irradiated wells are provided in Fig. S10,† for qualitative comparison. The results demonstrate the impact blue light may have on cancer cell cultures independently of the presence of any photopharmaceuticals, whereas green or red light doses lower than 38 J cm^–2^ can be considered as having a negligible impact on the growth of the cancer cell lines tested here.

**Table 3:** Effective doses (ED_50_) calculated from the light dose–response data of affected human cancer cell lines by fitting the curves using the two-parameter Hill-slope analysis with ±95% confidence intervals

Cell line	Wavelength (nm)	ED_50_ ^*a*^ (SRB assay)		
		Value (J cm^–2^)	+ Error	– Error
A375	455	10.9	3.7	2.8
	520	140	—	—
A549	455	30.5	12.5	8.9
MDA-MB-231	455	400	—	—
	520	350	—	—

^*a*^Positive and negative errors are not reported for ED_50_ values >100 J cm^–2^.

**Fig. 4:**
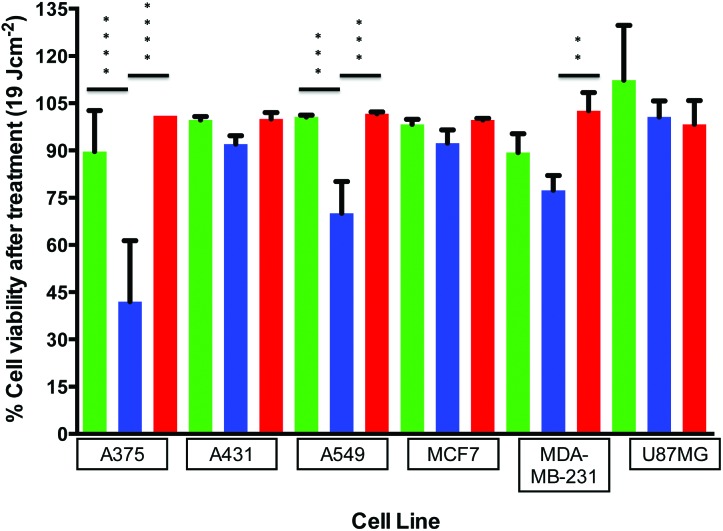
Comparison of cell viabilities (irradiated *vs*. non-irradiated) for each cell line at the same dose of 19 J cm^–2^ using blue (455 nm, 30 min, 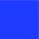), green (520 nm, 15 min, 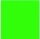), or red (630 nm, 9 min, 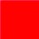) light. Averages of the cell viability percentages (blue and green irradiated or blue and red irradiated) were analyzed using a one-way ANOVA. **p = 0.005, ***p = 0.0005, ****p < 0.0001.

A further analysis of cell responses to blue, red, and green light at equivalent doses provides information about the specific effect of the wavelength of light. For example, 10 min of irradiation using the 455 nm LED array correlates to the same dosage (6 J cm^–2^) as 5 min irradiation using the 520 nm LED array or 3 min irradiation using the 630 nm LED array (Table 2). This correlation was also observed at a dose of 19 J cm^–2^ (30 min at 455 nm, 15 min at 520 nm, or 9 min at 630 nm). The differences between blue, green, and red light responses at identical doses were tested using an ordinary one-way ANOVA. Each set of sample averages at 19 J cm^–2^ (blue *vs*. green, blue *vs*. red, and green *vs*. red) was analysed for a specific cell line; the results are shown in Fig. 4, with the asterisks denoting significant differences between populations. As expected, no significant differences in the averages were observed between green and red for all cell lines. Additionally, for the cell lines A431, MCF7, and U-87 MG, which were unaffected by blue light, there were no significant differences between blue, green, or red averages. However, comparison of the blue *vs*. green or blue *vs*. red irradiation responses for the A375, A549 and MDA-MB-231 cell lines indicates differences in cell viability. The difference between blue (455 nm) and green (520 nm) irradiation is significant for A375 and A549, but not for MDA-MB-231. Comparing the blue (455 nm) to the red (630 nm) irradiation, all three cell line response averages are significant in the order of A375 > A549 > MDA-MB-231. It should be reiterated that these differential effects were not a result of varied thermal build-up during irradiation (Table 1). Thus, visible light damage is blue light-specific (or wavelength specific) for three out of the six cancer cell lines investigated.

**Fig. 5:**
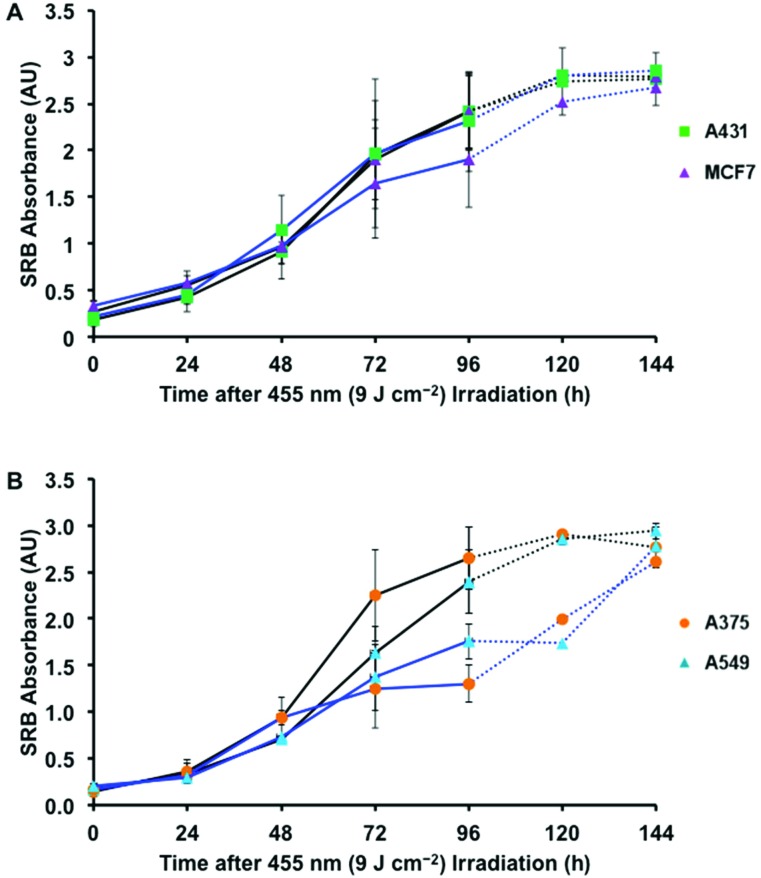
Cell growth curves for dark *vs*. blue light-irradiated samples (455 nm, 9 J cm^–2^) of unaffected (A) and affected (B) cancer cell lines. Solid lines represent 96 h experiments, dotted lines represent 144 h experiments (see text). Conditions: cells cultured and treated under the same conditions as in Fig. 4. Cells were fixed using TCA at 4, 24, 48, 72, and 96 h after seeding and then stained with SRB. The SRB absorbance of ten technical replicates (*n*_t_ = 10) was averaged for one experiment; three biological replicates were performed (*n*_b_ = 3).

In order to further understand the observed blue light irradiation effect, the growth curves of non-irradiated and irradiated cells were evaluated every 24 h until 96 h after seeding. A set of control growth curves in the dark was determined first to analyse the doubling times of each cell line using an exponential growth fitting (Fig. S11†). In such conditions the cells display typical growth curve characteristics, including the log (0–20 h), log (20–72 h), and stationary phase (>96 h). The doubling times of all the cell lines were between 20–40 h (Fig. S11C†). The effect of blue light on growth curves is shown in Fig. 5 (solid lines). As expected, the two unaffected cell lines tested, A431 and MCF7, did not display significant differences between blue light-irradiated samples and their dark controls (Fig. 5A). However, A375 and A549 cells displayed very different growth curves, as cell growth was inhibited at 48 h following irradiation (Fig. 5B). Strong growth inhibition was observed in A375 cells, followed by what appears to be a stationary phase or cytostasis. A549 cells displayed less inhibition initially, but at 96 h entered late-onset growth inhibition. To determine whether A375 cells were in a cytostatic state and whether A549 cells displayed further inhibition, the experiment was repeated and extended to 120 and 144 h for two affected (A375 and A549) and two unaffected (A431 and MCF7) cell lines (Fig. 5, dotted lines). At 120 and 144 h after irradiation, the unaffected cells continued to follow similar trends compared to control plates. The growth curve for A375 cells indicates that, after 96 h, the cells recovered by 144 h, but at a slower rate than the normal log phase. In contrast, A549 cells reached a later stage plateau at 120 h, but then recovered at a faster rate than the control log phase. Although it initially appeared that the A375 cells had become cytostatic, the data indicate that 96 h post irradiation these as well as A549 cells, fully recovered. For the A549 cells, the results suggest that cell proliferation increased after blue light irradiation.

## Discussion

**Fig. 6:**
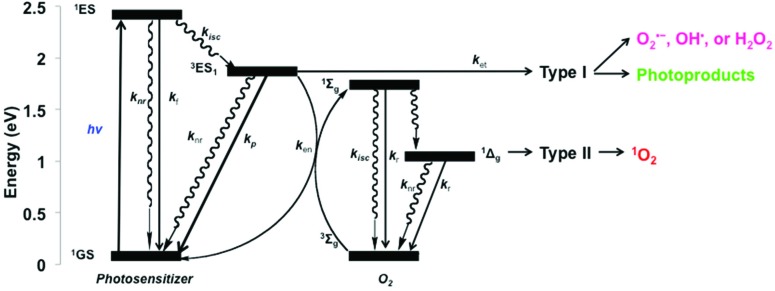
Generalized Jablonski diagram showing the possible outcomes following high-energy visible light excitation of endogenous photosensitizers with molecular oxygen and other cell substrates; rate constants (*k*) for f (fluorescence), p (phosphorescence), r (radiative), nr (non-radiative), isc (intersystem crossing), en (energy transfer), and et (electron transfer) are indicated.

Although the exact mechanism of action for blue light-induced cytotoxicity is currently elusive, general mechanisms of light–cell interactions are known. In normal cells, studies on the effects of HEVL have gained significant interest when attempting to understand the effects of prolonged, low-intensity exposure to blue light in relation to electronic device screens, curing dental materials, or the treatment of normal retinal,^26–33^ oral,^34,35^ and skin^36–38^ cells. For normal cells, endogenous chromophores may act in a similar manner as a photodynamic therapy (PDT) agent. PDT uses an exogenous photosensitizer to cause cell damage upon visible light irradiation, *via* two different mechanisms, type I or type II (Fig. 6). Upon irradiation certain chromophores can populate a long-lived triplet excited-state (^3^ES) that may undergo a type I electron transfer to form superoxide (O_2_˙^–^), which undergoes dismutation to other reactive oxygen species (ROS), *i.e.*, OH˙ or H_2_O_2_. Alternatively, a type II mechanism may occur, which involves energy transfer from ^3^ES to the ground state of molecular oxygen (^3^O_2_) to form singlet oxygen (^1^O_2_). Both type I and II mechanisms result in the oxidation of biomolecules and cellular oxidative stress.^39^ Additionally, both conditions may result in cell proliferation, cell cycle arrest, autophagy, mitophagy, and/or cell death, which depends on the extent of reactive intermediate formation, the location in which the reactive intermediates are formed,^40^ and the genotype/phenotype of the cell, among other factors. Following the blue light effect on normal cell studies, it was proposed that oxidative stress is induced as a result of endogenous chromophore excitation, resulting in type I electron transfer reactions.

Studies on the effect of high-energy visible light on cancerous cells are more limited.^34,35,41–49^ Ohara *et al.* showed that blue light (LED array, 470 nm, 6.8 J cm^–2^) caused >50% growth inhibition of B16 mouse melanoma cells grown in EMEM supplemented with 10% FCS.^45^ Irradiation of B16 cells under considerably harsher conditions (Waldman lamp, 380–470 nm, 20 J cm^–2^) in the presence of DMEM supplemented with 10% FCS resulted in >85% cytotoxicity, but no lipid peroxidation.^47^ In the most recent paper regarding visible light toxicity in human cancer cells, Matsumoto *et al.* showed that HT29 and HCT116 human colorectal cancer cell lines grown in RPMI 1640 medium supplemented with 10% FCS were strongly affected by blue wavelength light (465 nm, 30 mW, 10 min per day for 5 days), but not green (525 nm) or red (635 nm) under the same power and time conditions.^48^ The irradiated HT29 and HCT116 cells exhibited approximately 20–30% viability compared to non-irradiated cell populations. Further cell cycle and mRNA expression analyses were performed in HT29 cells, which revealed that the blue light-irradiated cells were mostly in the sub-G_1_ phase (apoptotic), exhibited increased caspase 3 and caspase 9 activity, and up-regulated Fas death receptor and Jun N-terminal kinase (JNK) in comparison with non-irradiated samples. According to ref. 48 such results suggest that apoptosis is induced *via* an external pathway.

Due to the wide variations in experimental setups, the previously published results on light-induced effects on cell viability are difficult to compare. These differences, nevertheless, demonstrated that *in vitro* blue light doses as low as 20 J cm^–2^ induced significant cell death, which correlates well with our work. However, we observed a blue light response in certain cancer cell lines, but no effects in other types of cancer cells subjected to the exact same conditions. Although previous reports focused on a single cancer cell line or a cell line from a specific organ, we found interesting differences in blue light response between cancer cell lines from similar organ origins (*e.g.* skin: A375 *vs*. A431) as well as from different origins (*e.g.* lung *vs*. brain). The fact that cancer cells are typified by several genetic mutations compared to normal cells makes it difficult to deduce the exact mechanism of blue light toxicity for each cell type. Due to the wavelength specificity of blue light-induced cancer cell damage (400–500 nm), it has been proposed that chromophores in cancer cells or in the media, such as flavins (*λ*_abs_ = 400–500 nm) and/or porphyrins (*λ*_abs_ = 400–650 nm) may be the reason for HEVL toxicity.^27,34,42,45,46,50–53^ However, the concentrations of flavins and porphyrins in cancer cells compared to normal cells is debatable with very few direct or significant comparisons available.^23,54–56^ Additionally due to the variety of media compositions, determining the specific chromophore cocktail makes it difficult to discern which compound is responsible for the blue light-specific cell death.^52,53^

Finally, the variable genetic mutations found in cancer cells lead to significant differences in phenotypes, such as circumvention of programmed cell death, up-regulated proliferation, recovery *via* autophagy, and/or increased tolerance to oxidative stress.^57–62^ The different phenotypes might also modify the intracellular signalling response to external stimuli, such as blue light or oxidative stress induced by internal or external dye excitation. From previous blue light-induced cytotoxicity studies and from the results presented here, we noticed interesting similarities. The human cell lines that displayed blue light toxicity (HT29, HCT116, A375, A549, and MDA-MB-231) also have mutations in their mitogen-activated protein kinase (MAPK) pathways (protein kinases that are specific for serine, threonine, and tyrosine). Specifically, the mutations occur in the protein sequences of the KRAS or BRAF families, which are responsible for growth, proliferation, differentiation, and apoptosis (Table S1†).^63–65^ The mutations are implicated in UVA and blue light mediated cancer mutagenesis as well as UVA stimulated light response mechanisms.^66–68^ Thus, such mutations could result in the specificity observed for blue light-induced cytotoxicity and/or recovery in these cell lines. Although some insight was provided by the mRNA expression analysis of the HT29 cell line,^48^ further analyses with a larger panel of cell lines will be necessary to fully understand the impact of blue light on cancer cell lines, and whether there is a better way to predict blue light sensitivity.

## Experimental

### Materials

The cell irradiation system consists of a thermostat (Ditabis Digital Biomedical, P/n: 980923001) fitted with two flat-bottom microplate thermoblocks (Ditabis Digital Biomedical, P/n: 800010600) and a 96-LED array fitted to a standard 96-well plate (1 LED per well). The LEDs (455 nm, FNL-U501B22WCSL; 530 nm, OVL-3324; 630 nm, OVL-3328), fans (40 mm, 24 V DC, 9714839), and power supply (EA-PS 2042-06B) were ordered from Farnell. The printed circuit boards (PCB) were from euroCircuits. Cells (A375, human malignant melanoma; A431, human epidermoid carcinoma; A549, human lung carcinoma; MCF7, human mammary gland adenocarcinoma; MDA-MB-231, human mammary gland adenocarcinoma; U-87 MG, human glioblastoma-grade IV) were distributed by the European Collection of Cell Cultures (ECACC), but purchased through Sigma Aldrich. Dulbecco's Modified Eagle Medium (DMEM, with and without phenol red, and without glutamine), 200 mM glutamine-S (GM), trichloroacetic acid (TCA), glacial acetic acid, sulforhodamine B (SRB), and tris(hydroxymethyl)aminomethane (tris base) were purchased from Sigma Aldrich. Fetal calf serum (FCS) was purchased from Hyclone. Penicillin and streptomycin were purchased from Duchefa and were diluted to a 100 mg per mL penicillin/streptomycin (P/S) concentration. Trypsin and Opti-MEM (without phenol red) were purchased from Gibco Life Technologies. Trypan blue (0.4% in 0.81% sodium chloride and 0.06% potassium phosphate dibasic solution) was purchased from BioRad. Plastic disposable flasks and 96-well plates were from Sarstedt.

### Methods

#### LED setup

The LED setup was designed to irradiate one 96-well plate with visible light while maintaining a second twin plate as dark control at controlled temperature (Fig. 1A). A Ditabis thermostat fitted with two flat-bottom microplate thermoblocks was used to provide thermal control of the dark and irradiated plates (see the ESI†). Three LED arrays were custom-built by the Departments of Fine Mechanics and Electronics at Leiden University. Printed circuit boards (PCBs) for each LED array were patterned as 12 columns and eight rows (Fig. S1†) corresponding to the 96-well plate configuration. Each column of eight LEDs was wired in series. Two 100 Ω resistors were added to the eight LEDs in series. Where necessary, one calibration resistor was placed in parallel to one 100 Ω resistor in order to improve equality of light intensity. The 12 LED columns were wired in parallel (Fig. S2†). Each array (455 nm, 520 nm, and 630 nm) was fitted into an external block and a small fan was positioned in the centre to avoid over-heating. Variable resistors were added to control fan speed. The average height of each LED was 13–14 mm above the bottom of each well. The viewing angle (2*θ*_1/2_) for the blue, green, and red LEDs was 25°, 30°, and 30°, respectively. For the “dark” plate an external block was constructed without LEDs or fans. All LED blocks were manufactured with slits at the ends to allow airflow (Fig. S3†). A single LED array was driven using a standard power supply at constant voltage, which in principle allows for modulating light intensity. However, to minimize the number of parameters for a given experiment, the voltage was kept constant at 28.9, 27.9, and 20.7 V for the blue, green, and red light arrays, respectively. Under these conditions, light intensity was measured using chemical actinometry (where possible) and a physical sensor (integrating sphere) to crosscheck the observed values (see the ESI†). The properties of the arrays are summarized in Table 1. For dose calculations (J cm^–2^, Table 2), the power density measured using the integrating sphere (W cm^–2^) was multiplied by the irradiation time (s). In all biological experiments the doses were calculated based on the power density measured by the integrating sphere.

#### General cell culturing

Cells were cultured in DMEM complete (Dulbecco's Modified Eagle Medium (DMEM) with phenol red, supplemented with 8.0% v/v fetal calf serum (FCS), 0.2% v/v penicillin/streptomycin (P/S), and 0.9% v/v glutamine-S (GM)). Cells were cultured under our standard culturing conditions (humidified, 37 °C atmosphere containing 7.0% CO_2_) in 75 cm^2^ flasks and subcultured (1 : 3 to 1 : 6 ratio) upon reaching 70–80% confluency (approximately once per week). Medium was refreshed every second day. Cells were passaged for 4–8 weeks.

### Determination of light-induced cell death

#### Preparation of cell culture samples

The photocytotoxicity of blue, green, and red light irradiation was assessed in six human cancer cell lines according to the following method. Cells from the general culturing conditions were detached by trypsinization, DMEM complete was added for trypsin deactivation, and cells were pelleted by centrifugation. The cell pellet was re-suspended in Opti-MEM (without phenol red) supplemented with 2.4% v/v FCS, 0.2% v/v P/S, and 1.0% v/v GM (Opti-MEM complete). Cells were stained using a 1 : 1 ratio of cell suspension : trypan blue, counted using a BioRad TC10 automated cell counter, and diluted to the appropriate seeding density. The seeding density was 7 × 10^3^ (A375), 8 × 10^3^ (A431), 5 × 10^3^ (A549), 8 × 10^3^ (MCF7), 1.2 × 10^4^ (MDA-MB-231), and 6 × 10^3^ (U-87 MG) cells per well (100 μL volume).

All cells were seeded at 0 h, irradiated at 48 h, and assayed at 96 h (Fig. 2A). Each cell line was seeded in a row of ten wells (Fig. 2B) per plate. “Dark” and “irradiated” plates were run concomitantly for each of the four different time points. After seeding, cells were incubated in the dark for 24 h under a humidified, 37 °C atmosphere containing 7.0% CO_2_. After 24 h incubation, 100 μL of Opti-MEM complete was added as a mock photochemotherapeutic drug treatment; the cells were incubated for an additional 24 h.

#### Light irradiation of cells

At 48 h, cells were irradiated. The medium was refreshed using Opti-MEM complete and twin plates were placed in the dark or irradiation compartments of the 96-well LED array system (Fig. 1A). The thermoblock was set to 39 °C and preheated for at least 10 minutes, resulting in an in-well temperature of 35–37 °C (see the ESI†). The plates were either kept under dark control conditions or irradiated for 5, 10, 15, or 30 min at 455 or 520 nm, or 3, 6, 9, or 18 min at 630 nm (see Fig. 1B for LED spectra and Table 2 for light dosage). Following irradiation, cells were cultured for another 48 h under standard conditions.

#### SRB endpoint assay

At 96 h after seeding, the relative cell viability was determined using the sulforhodamine B (SRB) assay.^69^ Briefly, cells were fixed using 100 μL of cold trichloroacetic acid (TCA, 10% w/v) and maintained at 4 °C for 4–48 h. Next, TCA was removed from the wells, the plates were gently washed five times with water, air-dried, stained using 100 μL sulforhodamine B (0.6% w/v SRB in 1% v/v acetic acid) for 30–45 minutes, washed five times with ∼300 μL acetic acid (1% v/v), and air-dried. The dye was then solubilized using 200 μL of tris base (10 mM). The absorbance in each well was read at 510 nm using a M1000 Tecan Reader.

#### Cell viability data analysis

The SRB absorbance data were used to calculate the fraction of viable cells in each well using Excel and GraphPad Prism. The absorbance data from ten wells (technical replicate, *n*_t_ = 10) for each cell line were averaged. Relative cell viabilities were calculated by dividing irradiated sample absorbance by the absorbance of the dark control. Three biological replicates (*n*_b_ = 3) were completed for each wavelength and cell line. For each biological replicate, cells were assigned to different rows to reduce sample bias. The average cell viability of the three biological replicates was plotted *vs*. log(light dose in J cm^–2^) with standard deviation error of each point. Using the light dose–response data for each cell line, the ED_50_ (effective light dose) was calculated by fitting the curves using a non-linear regression function with fixed *Y* maximum (100%) and minimum (0%) (relative cell viability), and a variable Hill-slope, resulting in the simplified two-parameter Hill-slope eqn (1).1
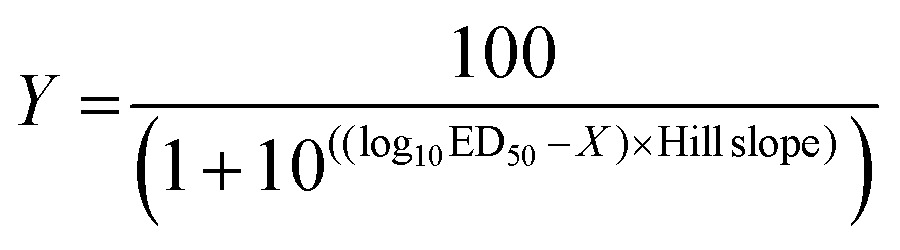



The difference between the blue, green, and red light cell response averages at a dose of 19 J cm^–2^ was compared by one-way ANOVA. For each cancer cell line, the average over three biological replicates of the relative cell viability at 19 J cm^–2^ was analyzed.^70^ The averages of blue *vs*. green, blue *vs*. red, and green *vs*. red responses for all cancer cell lines were compared. A *P*-value ≤0.05 was considered statistically significant.

### Effect of blue light irradiation on cell proliferation

To compare experimental growth curves after blue light irradiation to that in the dark, cells were seeded at *t* = 0 and an additional 100 μL of media was added at *t* = 24 h (mock treatment). At *t* = 48 h, cells were irradiated or left in the dark as control. At 4, 24, 48, 72, 96, 120, and/or 144 h after seeding (Fig. 2A, grey outline) the cell viabilities were determined using the SRB assay (see above). For the 4 h and 24 h time points (*i.e.*, before irradiation) a single plate was seeded, and for the remaining time points “dark” and “irradiated” plates were run in parallel. At 48 h, after seeding the plates in the irradiated group were treated with blue light for 15 min (455 nm, 10.5 ± 0.7 mW cm^–2^), corresponding to a light dose of 9 J cm^–2^. The SRB absorbance data from ten wells (*n*_t_ = 10) for each cell line were averaged and used to differentiate between the dark and irradiated growth curves. The doubling time was analysed using the GraphPad Prism exponential curve analysis. Three biological replicates were completed (*n*_b_ = 3) for each cell line.

## Conclusions

We have developed a LED irradiation system for photochemical and photobiological testing, which is economical, fully characterized, and reliable. In addition, we have provided a standardized biological testing protocol compatible with visible light irradiation and that uses the SRB assay as an endpoint assay. The SRB assay is used by the National Cancer Institute for their high-throughput drug testing and is generally considered a standard assay for determining cell populations. With the irradiation system and protocol in hand, we tested the effect of visible light on cancer cell lines, and showed an example of PDT dye testing *in vitro*.

The general consensus in the photo(chemo)therapeutic community is that UV irradiation is harmful to cancerous and non-cancerous cells and therefore should be avoided, whereas light in the visible spectrum is considered non-toxic and better suited for the activation of photopharmaceutical compounds in tissues (cancerous or non-cancerous). However, control experiments with illumination in the absence of photopharmaceuticals are often not shown. Our results show that blue light alone can lead to significant reduction in cell population, and that reporting cell viabilities following illumination without any compound is a critical control in the assessment of the photopharmacological properties. The present study revealed that the effect of blue light on human cancer cells at a dose of 19 J cm^–2^ depends on the cell line and is specific for 455 nm wavelength light, with green light (520 nm) and red light (630 nm) showing a negligible impact on the six cell lines tested at comparable light doses. At 9 J cm^–2^ blue light exposure, the light-sensitive cell lines (A375 and A549) exhibited temporary cessation of proliferation, which was ensued by a pro-proliferative response 96 h post-irradiation. It should be mentioned that due to the limitations of the SRB assay only the cell quantity was measured, rather than the cell quality so no specific information about the mechanistic foundation of these effects was determined. In light of the many possible mechanisms postulated above, more extensive photobiological investigations are required to determine the selective sensitivity of certain cancer cell lines to blue light.
