# Supplementary material for: An in vitro cell irradiation protocol for testing photopharmaceuticals and the effect of blue, green, and red light on human cancer cell lines
Source: Photochem Photobiol Sci. 2016 Mar 31;15(5):644–53. doi: 10.1039/c5pp00424a (PMC5044800; doi:10.1039/c5pp00424a)
Supplement: Supplementary file 1 [file PP-015-C5PP00424A-s001.pdf]

In vitro cell irradiation protocol for testing photopharmaceuticals and the effect of blue, green, and red light on human cancer cell lines

Samantha L. Hopkins,[a] Bianka Siewert,[a] Sven Askes,[a] Peter Veldhuizen,[b] Raphaël Zwier,[b] Michal Heger,[c] and Sylvestre Bonnet *[a]

[a] Leiden Institute of Chemistry, Leiden University, Einsteinweg 55, 2300RA Leiden, The Netherlands
[b] Leiden Institute of Physics, Leiden University, Niels Bohrweg 2, 2333CA Leiden, The Netherlands
[c] Department of Experimental Surgery, Academic Medical Center, University of Amsterdam, Meibergdreef 9, 1105 AZ Amsterdam
Corresponding author email: bonnet@chem.leidenuniv.nl

Supplementary Information

Table of Contents

Table of Contents 1

I. Materials 2

II. LED irradiation setup 2
- **Figure S1**: Diagram of the printed circuit board (PCB) used for each LED array. 2
- **Figure S2**. Schematic of the electronics of the 455, 520 and 630 nm LED arrays. 3
- **Figure S3**. External cover dimensions and rendering for LED arrays. 4

III. LED power density setup and analysis, Integrating sphere 5
- **Figure S4**. Lab-built integrating sphere setup for measuring the irradiance of the LED array in ten wells (A) and birds-eye view of integrating sphere located at well D4 (B). ... 6

IV. LED power density in wells using chemical actinometry 6
- **Figure S5**. Fe(II) calibration curve (A) and a plot of the Fe(II) concentration (mol) vs. time irradiated (s) (B). 7
- **Figure S6**. Example of actinometry absorbance results for 15 s irradiation at 520 nm.. . 8

V. LED array thermal control 8

VI. Effective concentrations (EC_{50}) values for rose bengal and methylene blue 9
- **Figure S7**. Electronic absorption spectra of rose bengal and methylene blue in water at room temperature. 9
- **Figure S8.** Summary of EC_{50} data for rose bengal (RB) and methylene blue (MB) with 95% CI.. 11
- **Figure S9**. Example dose-response curves for rose-bengal treated A549 cells (A) and methylene blue treated A375 cells (B). 12

VII. Microscopy images of cells: 24 h, 48 h, 96 h (dark or irradiated) 13
- **Figure S10**. Micrographs (20X) of each cell line at 24 h, 48 h, and 96 h after seeding for dark populations and at 96 h for blue, green, or red irradiated cells (19 $J \cdot cm^{-2}$). 13

VIII. Growth curve in the dark 14
- **Figure S11**. Growth curve (A), fitted exponential growth curves (B), and doubling times with 95% confidence interval (C) for A375 (orange), A431 (green), A549 (blue), MCF7 (purple), MDAMB231 (pink), and U-87 MG (gray) using SRB cell counting assay.. 14

IX. Cancer cell line gene mutation information 15
- **Table S1.** Cancer cell line with gene mutations and if available the protein sequence.[7] 15

I. Materials

The integrating sphere (AvaSphere-30-IRRAD), spectrometer (AvaSpec-ULS2048L StarLine CCD spectrometer), calibrated optical fiber (FC-UV600-2 optical fiber), and calibration lamp (AVALIGHT-HAL-CAL-ISP30) were from Avantes (Apeldoorn, The Netherlands). The integrating sphere setup was constructed from poly(methyl methacrylate) sheets (Perspex®) and poly(acetyl) copolymer rods (Delrin®). $FeCl_3$ and $K_2C_2O_4 \bullet H_2O$ for synthesizing the ferrioxalate chemical actinometer were from Alfa Aesar and Acros. The standard 0.05 and 0.5 N sulfuric acid solutions were made from Fluka Analytical FIXANAL® concentrate from Sigma Aldrich. Ammonium iron(II) sulfate hexahydrate was from Sigma Aldrich. The phenanthroline developing solution was made from sodium acetate trihydrate and 1,10-phenanthroline monohydrate from VWR and Alfa Aesar. The thermocouple was from Omega.

II. LED irradiation setup

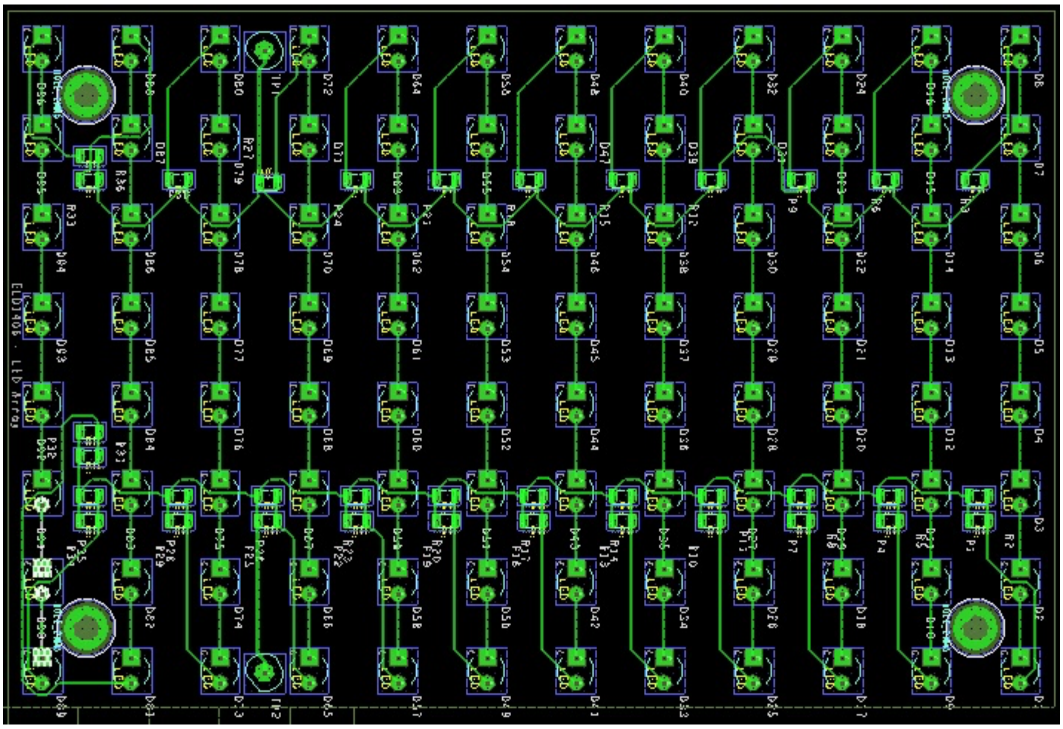

Figure S1: Diagram of the printed circuit board (PCB) used for each LED array.

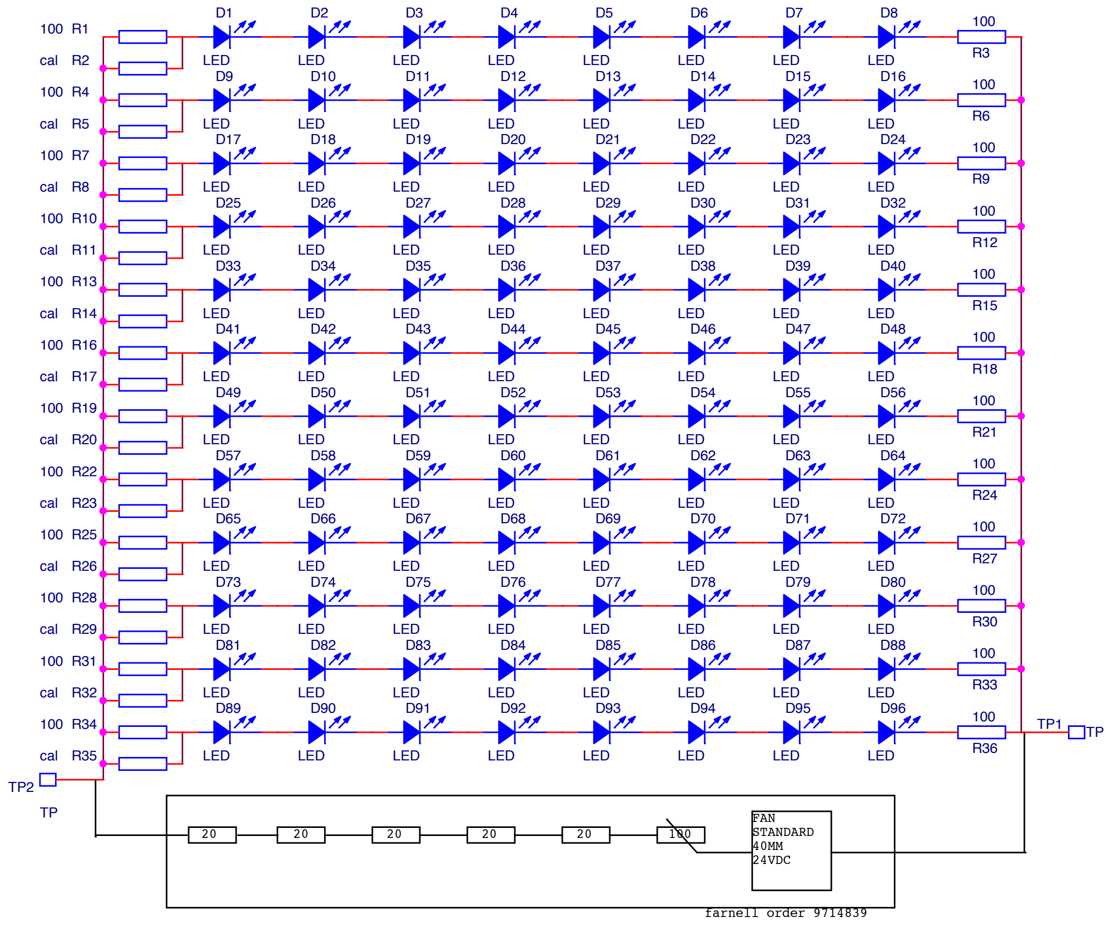

Figure S2. Schematic of the electronics of the 455, 520 and 630 nm LED arrays.

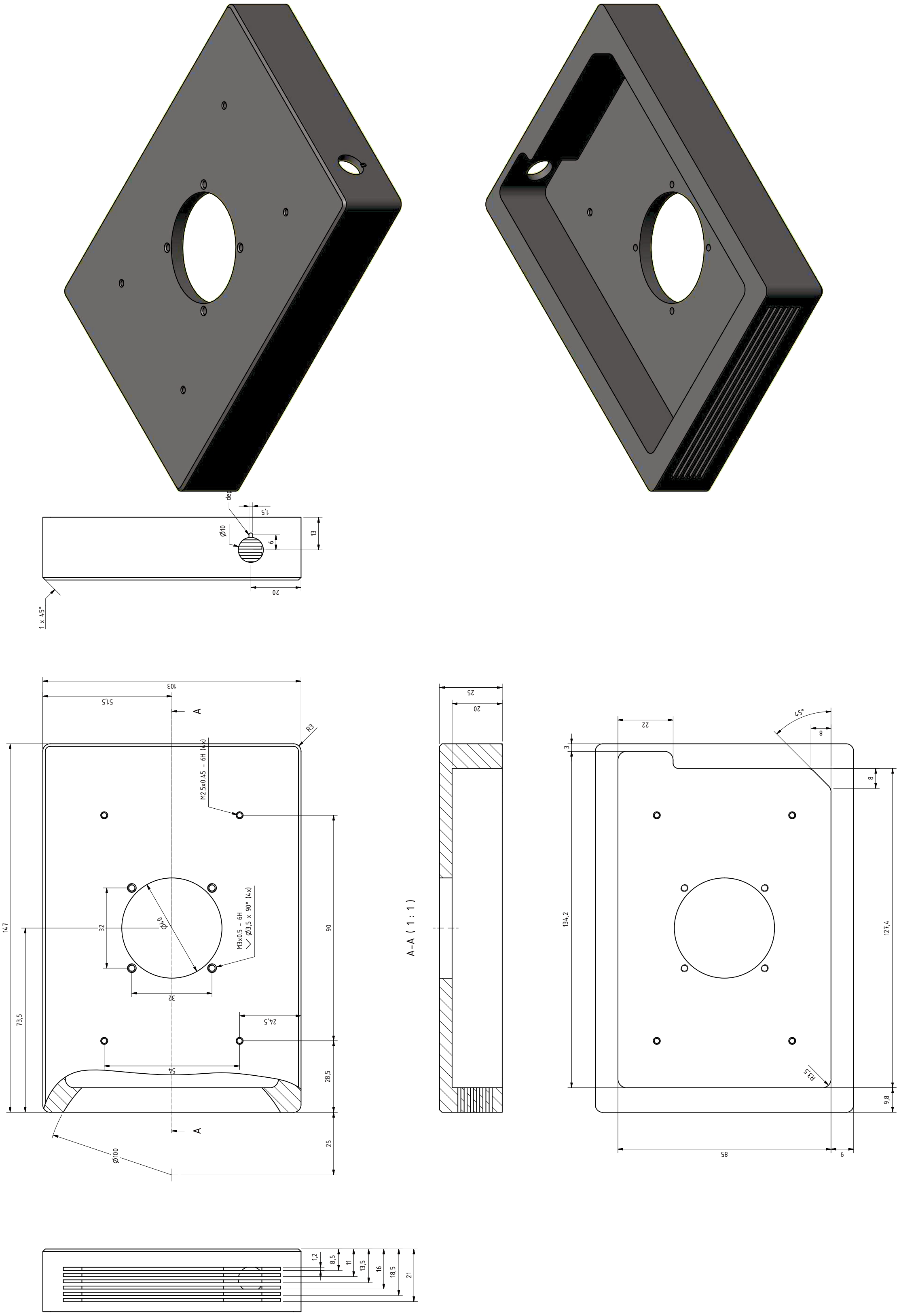

Figure S3. External cover dimensions and rendering showing the location of the fan, lateral slits for air circulation, plug input and diagonal cut for keeping the plate in place for LED arrays.

III. LED power density setup and analysis, Integrating sphere

The light power density of the LED arrays was measured either using a lab-built setup using physical sensing, or chemical actinometry. The physical sensing setup consisted of an integrating sphere, which was positioned underneath the 96-wells plate to simulate the irradiation conditions during cell experiments (Figure S4A). The integrating sphere was mounted in a custom-made holder that aligned the 6 mm aperture of the integrating sphere with the 6 mm diameter of a single well in a 96-wells plate, while the LED array was placed on top of the well plate (with its lid), like during cell irradiation. The integrating sphere position was adjustable over ten wells thus providing a diagonal representative set of wells that could be measured individually. The integrating sphere was connected *via* an optical fiber to an Avantes CCD spectrometer. The spectrometer, in combination with the sphere and fiber, were spectrally calibrated directly before measurement, using an Avantes calibration lamp, to report the spectrum in absolute irradiance units ($\mu W{\cdot}cm^{-2}{\cdot}nm^{-1}$), where the surface here refers to that of the aperture of the integrating sphere. The power supply was set to the appropriate voltage, 29.9, 27.9, or 20.7 V for the 455, 520, or 630 nm LED arrays, respectively. The integrating sphere was then placed in the setup and centered under a specific well, for example D4 (Figure S4B). The LED system was then placed on top of the setup and an average of several scans was measured. These steps were repeated for all ten wells (B2, C3, C7, D4, D8, E5, E9, F6, F10, and G11). An average from the ten data points was taken to determine the values for the entire array. The emission maximum and full width at half maximum (FWHM) bandwidth for each LED array wavelength was determined from the emission spectra (Table 1). Using Excel and OriginPro, the total power density at the bottom of each well ($mW{\cdot}cm^{-2}$) was calculated by integrating the area under the spectral irradiance *vs*. wavelength curve, multiplying by the surface area of the integrating sphere aperture (diameter = 6 mm, surface 0.28 cm^2), and dividing the resulting power (mW) by the surface area of a well (0.32 cm^2). Average power densities and standard deviations were 10.5 ± 0.7, 20.9 ± 1.6, and 34.4 ± 1.7 $mW{\cdot}cm^{-2}$ for the 455, 520, and 630 nm LED arrays, respectively (Table 1). Using an integrating sphere provides a straightforward physical method to test the LEDs, but assumes that the single LED measured per 8-LED column is not significantly different from the remaining seven. Therefore, chemical actinometry (in all 96-wells) was used as a comparison for the blue and green LED arrays. **Note**: it was impossible to find a chemical actinometer for red light compatible with aqueous solutions, so this comparison was not done for 630 nm.

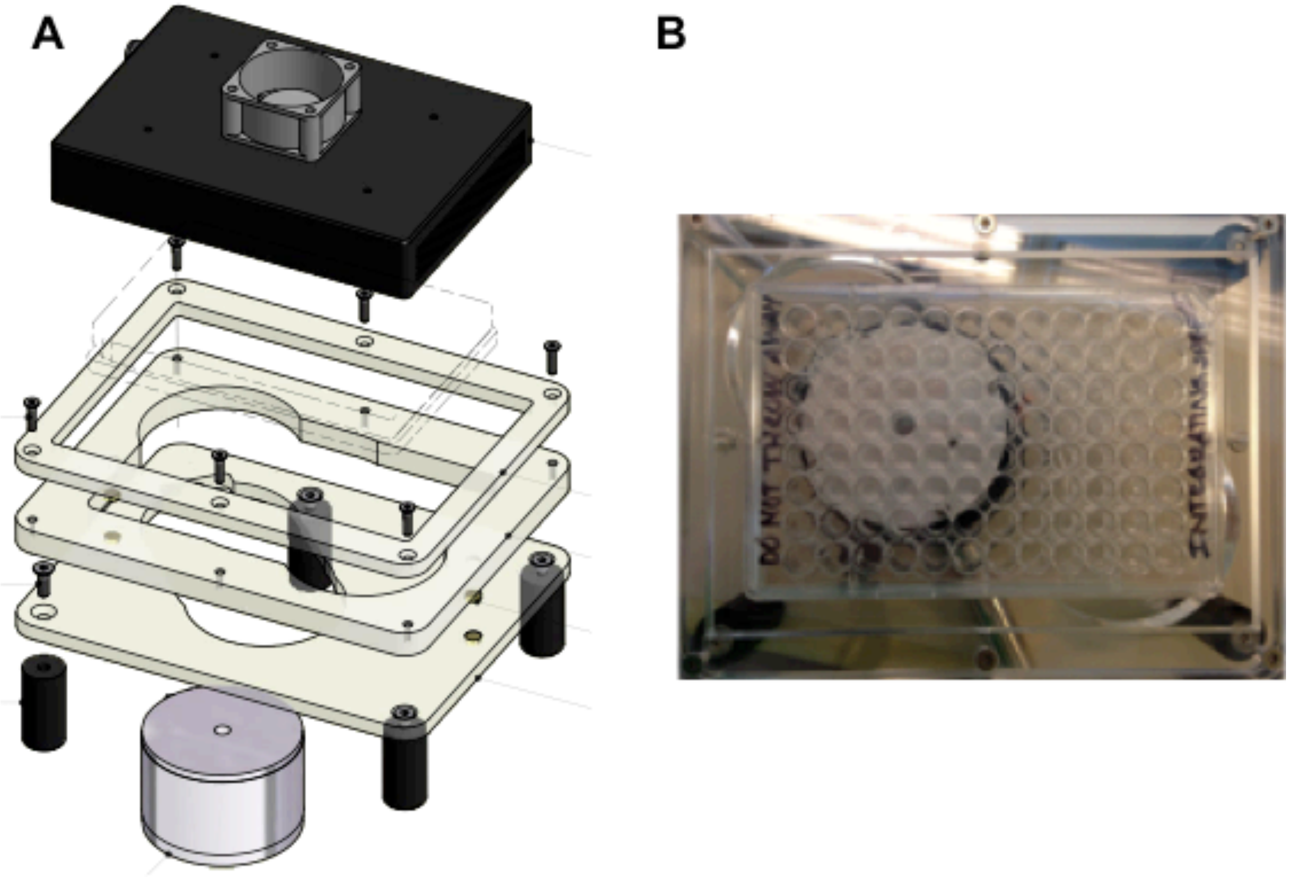

Figure S4. Lab-built integrating sphere setup for measuring the irradiance of the LED array in ten wells (A) and birds-eye view of integrating sphere located at well D4 (B).

IV. LED power density in wells using chemical actinometry

A chemical actinometer measures the number of photons absorbed into a defined volume, in this case 200 µL aqueous solution in a well of a 96-wells plate, per unit time.[1] The ferrioxalate actinometer ($K_3[Fe(C_2O_4)_3]$) and phenanthroline-based developing solutions were made using a previously published method.[2-4] Briefly, a 0.015 M or 0.15 M ferrioxalate solution in 0.05 N sulfuric acid was prepared for 455 nm or 520 nm irradiation, respectively. To the central 60 wells, 200 µL of actinometer solution was added. “Dark” and “irradiated” plates were run in parallel. Plates were irradiated for 10, 15, 20, 25, or 30 s (455 nm) or 15, 30, 45, 60, or 75 s (520 nm). Following irradiation, 175 µL of the irradiated actinometer solution was removed and 325 µL of the phenanthroline development solution (1 g/L 1,10-phenanthroline buffered with 23 g/L sodium acetate trihydrate in 0.5 M sulfuric acid) was added. The absorbance at 510 nm was measured for each well using a M1000 Tecan Reader. The absorbance of the 60 wells was averaged for each time point. The absorbance values for the actinometry were converted to the number of mol of

$[Fe(phen)_3]^{2+}$, i.e., to the amount of Fe^{2+} ions produced during irradiation, using the calibration curve shown in Figure S5A, which was developed specifically for this protocol as follows. 25 µL of 0, 30, 45, 60, 75, 100, and 180 µM $[(NH_4)_2Fe(SO_4)_2{\bullet}6H_2O]$ in 0.05 N sulfuric acid were added to each column (eight wells), which was developed using 325 µL of the phenanthroline development solution. The absorbance at 510 nm *vs*. concentration (M) of Fe(II), was plotted, resulting in a linear fit with the equation S1 and R^2 = 0.995.

$$Y = 9538x + 0.05 \qquad \text{Equation S1}$$

The plot of the number of mol of Fe^{2+} produced *vs*. irradiation time (s) for the 455, 520, and dark setups are shown in Figure S5B. The slope of the data (Δmol $Fe^{2+}/\Delta t$) for each wavelength was converted to $Einstein{\cdot}cm^{-2}{\cdot}s^{-1}$ or the light flux for the sample by dividing the slope by the surface area of the well (A = 0.32 cm^2), quantum yield of ferrioxalate actinometer (Φ = 1.1 at 0.015 M and Φ = 0.65 at 0.15 M), and probability of ferrioxalate absorption at the specific wavelength of irradiation ($1\text{-}10^{-A}$). Finally the equivalent power density ($J{\cdot}cm^{-2}{\cdot}s^{-1}$ or $W{\cdot}cm^{-2}$) was calculated by multiplying the photon flux values in $Einstein{\cdot}cm^{-2}{\cdot}s^{-1}$ by the energy of a mol of photons at the central wavelength of the LED ($J{\cdot}Einstein^{-1}$). An average of the power density calculations was taken. The actinometry experiments (60 wells per time point) were repeated three times and an overall average was derived.

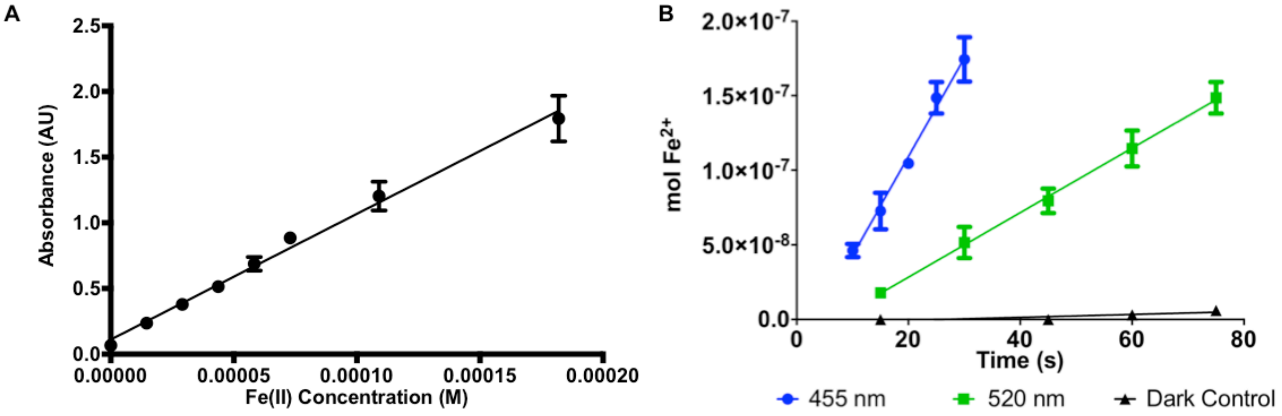

Figure S5. Fe(II) calibration curve to determine the absorbance as a function of Fe(II) concentration for a 350 uL volume in a well of a 96-well plate (A) and a plot of the Fe(II) concentration (mol) vs. time irradiated (s) for 455 nm (blue circle), 520 nm (green square), and dark systems (black triangle) (B).

Only the central 60 well absorbance values were used for averaging the power density because the values for the 36 outer wells of the irradiated 96-wells plate were approximately 75-80% of the absorbance measured for the central wells with increased standard deviation (> 20%, see Figure S6). These border wells excepted, the central 60 wells show uniform plate irradiation within 10% standard deviation. Dark controls were

used to verify that cells maintained in the dark were receiving negligible photon flux Figure S5B). The power densities using chemical actinometry for the LED arrays were 10.2 ± 0.9 mW·cm^{-2} (455 nm) and 16.6 ± 1.3 mW·cm^{-2} (520 nm). The 455 nm power density values obtained by actinometry and integrating sphere correlate very well, which validates the physical sensing method using the integrating sphere. By contrast the 520 nm measurement slightly deviates (Table 1), which is attributed to the very low absorbance of the ferrioxalate actinometer at 520 nm. 520 nm actually represents the ultimate wavelength at which the ferrioxalate actinometer can be used. As stated above, an additional limitation of chemical actinometry, is the incompatibility of chemical actinometers for red light with plastic plates, therefore no comparison could be done between the two methods at 630 nm. In the following, it is the power density values measured using the integrating sphere that was used for all dose calculations during blue, green, or red light irradiation experiments.

<>	1	2	3	4	5	6	7	8	9	10	11	12
A	0.19	0.22	0.25	0.28	0.23	0.24	0.21	0.30	0.21	0.23	0.23	0.19
B	0.21	**0.28**	**0.37**	**0.34**	**0.30**	**0.31**	**0.31**	**0.33**	**0.31**	**0.36**	**0.32**	0.20
C	0.22	**0.28**	**0.31**	**0.35**	**0.27**	**0.30**	**0.38**	**0.37**	**0.29**	**0.36**	**0.27**	0.26
D	0.27	**0.34**	**0.29**	**0.35**	**0.34**	**0.30**	**0.38**	**0.30**	**0.31**	**0.36**	**0.24**	0.23
E	0.28	**0.36**	**0.29**	**0.30**	**0.28**	**0.35**	**0.36**	**0.26**	**0.37**	**0.36**	**0.35**	0.18
F	0.26	**0.29**	**0.30**	**0.29**	**0.28**	**0.30**	**0.34**	**0.36**	**0.30**	**0.34**	**0.33**	0.23
G	0.31	**0.26**	**0.31**	**0.30**	**0.32**	**0.28**	**0.26**	**0.33**	**0.33**	**0.30**	**0.34**	0.26
H	0.23	0.32	0.28	0.28	0.30	0.25	0.25	0.32	0.34	0.34	0.30	0.24

< average	**average**	> average

Figure S6. Example of actinometry absorbance results for 15 s irradiation at 520 nm. The average absorbance at 510 nm of the central 60 wells was 0.32 ± 0.03 AU (gradient colors with black writing yellow = lowest value to orange = highest value). The average of the border values was 0.25 ± 0.04 AU, where values below average are shown in red text and above average in green text.

V. LED array thermal control

The temperature in well D6 was measured to determine the thermal effects of the Ditabis thermoblock, either in combination with the fans cooling for the LED array, or without the fans for the dark control. In each well of two 96-wells plates, 200 µL phosphate buffered saline (PBS) was added. A thermocouple was used to measure the temperature over time in both “dark” and “irradiated” systems. The temperature was measured every 5 minutes up to 45 minutes and each measurement series was repeated three times.

To maintain a temperature range between 35-37 °C, the target temperature of the Ditabis thermostat needed to be set to 39 °C and PBS was pre-warmed in a water bath

(37 °C). Under such conditions, the temperature in well D6 was stably maintained between 35.5 and 37.5 °C for the blue, green, red, and dark systems.

VI. Effective concentrations (EC_{50}) values for rose bengal and methylene blue

Two standard PDT dyes were chosen for evaluating the irradiating setup on a known light-activatable compound. Both compounds are affordable, easily accessible, reproducibly soluble, and well-known in the literature. Rose Bengal was used for both blue (455 nm) and green (520 nm) light irradiation and methylene blue was used for the red (630 nm) light irradiation. For these experiments, the light dose was kept constant at 6 $J.cm^{-2}$ for all wavelengths. Rose Bengal is a well known $^1O_2$ generator ($\Phi_{\Delta 1O2}$ = 0.75 in H_2O) with negligible absorption in the red (Figure S7).[5] Methylene blue is a blue dye that absorbs in the red and has been used in a variety of PDT and aPDT type I and II applications (Figure S7).[6] Effective concentrations (EC_{50} values in μM) and the 95% confidence intervals for rose bengal and methylene blue using the standardized conditions of this manuscript are reported in Figure S8.

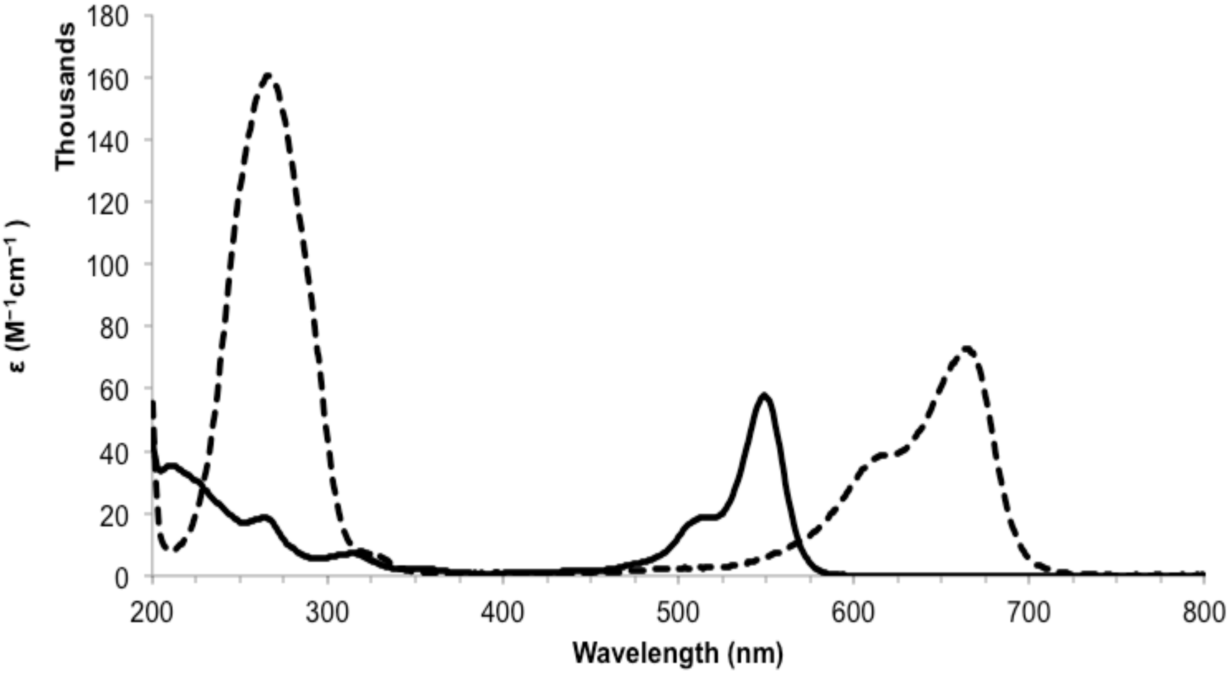

Figure S7. Electronic absorption spectra of rose bengal (solid line) and methylene blue (dashed line) in water at room temperature.

The conditions for these experiments were as follows: Cells were seeded at t = 0 at 7 x 10^3 (A375), 8 x 10^3 (A431), 5 x 10^3 (A549), 8 x 10^3 (MCF7), 1.2 x 10^4 (MDA-MB-231), and 6 x 10^3 (U-87 MG) cells/well in Opti-MEM complete without phenol red and incubated for 24 h at 37 °C and 7% CO_2. After one day the compounds were dissolved in DMSO (final ≤ 0.5%) and milliQ H_2O. Each compound was further diluted into a concentration series using Opti-MEM complete without phenol red and added to the cells at t=24 h. Cells were incubated in the presence of the compounds for another 24 h. At t=48 h media was removed and refreshed and then cells were irradiated at a dose of 6 J.cm^{-2} for blue (10 min), green (5 min), or red light (3 min). Following irradiation treatment, cells were placed back in the incubator for an additional 48 h. Cells were fixed using TCA at 96 h after seeding and then stained with SRB. The SRB absorbance of three technical replications was averaged for each experiment (n_b = 2).

The average absorbance for treated wells was divided by the untreated control well absorbance (n_t =6) to determine the relative cell viability. The average cell viability of the two biological replicates were plotted *vs.* Log(compound concentration in μM) with standard deviation error of each point. Using the light dose-response data for each cell line, the EC_{50} (effective concentration) was calculated by fitting the curves using a non-linear regression function with fixed Y maximum (100%) and minimum (0%) (relative cell viability), and a variable Hill-slope, resulting in the simplified two-parameter Hill-slope equation. Example dose-response curves are shown in Figure S9.

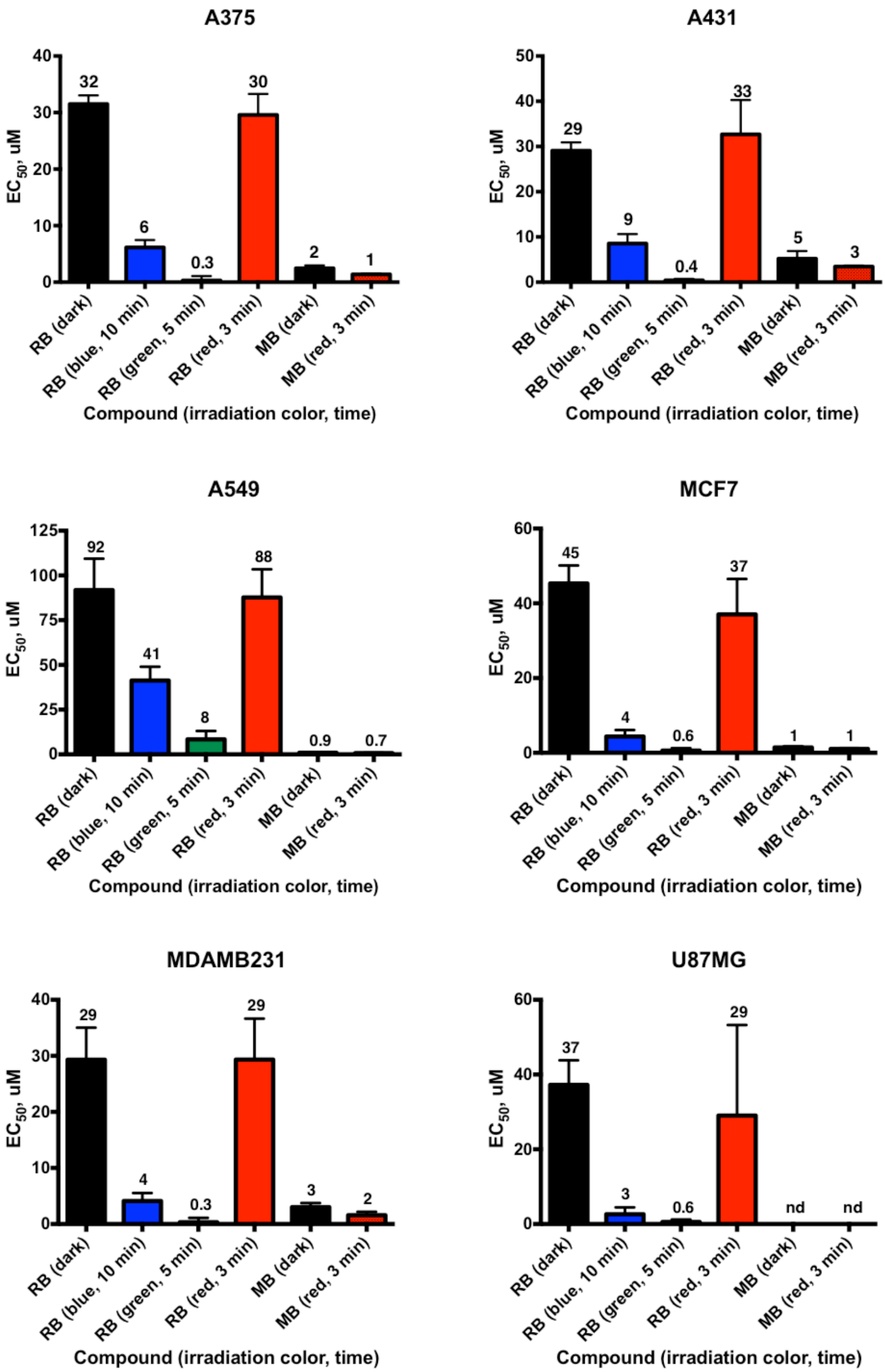

Figure S8. Summary of EC_{50} data for rose bengal (RB) and methylene blue (MB) with 95% CI. EC_{50} is reported above the bar for clarity, unless not determined (nd). For blue (455 nm) light irradiation, the irradiated controls were compared to the dark controls resulting in 60% (A375), 98% (A431), 100% (A549), 88% (MCF7), 86% (MDAMB231), and 99% (U87MG) viability. For green (520 nm) and red (630 nm) light irradiation, the irradiated controls were compared to the dark controls resulting in 95-100% viability for all cell lines.

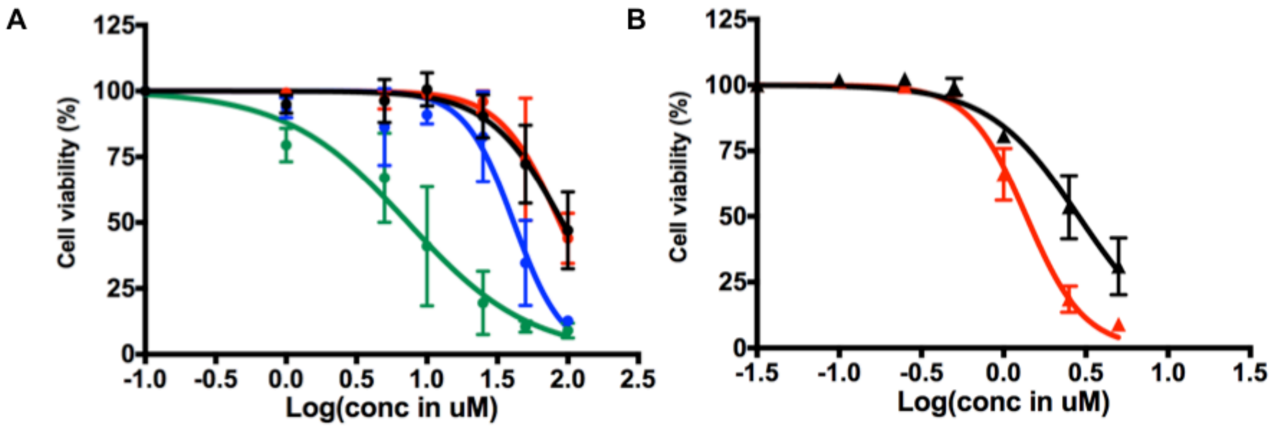

Figure S9. Example dose-response curves for rose-bengal treated A549 cells (A) and methylene blue treated A375 cells (B). Figure S8A shows the dose-response curves for dark (black circles) as well as blue (blue circles, 455 nm, 6 $J.cm^{-2}$), green (green circles, 520 nm, 6 $J.cm^{-2}$), and red (red circles, 630 nm, 6 $J.cm^{-2}$) irradiated responses. Methylene blue samples were dark treated (black triangles) and only irradiated using red light (red triangles, 630 nm, 6 $J.cm^{-2}$). The irradiated/light control cytotoxicity values for A and B were 100%.

VII. Microscopy images of cells: 24 h, 48 h, 96 h (dark or irradiated)

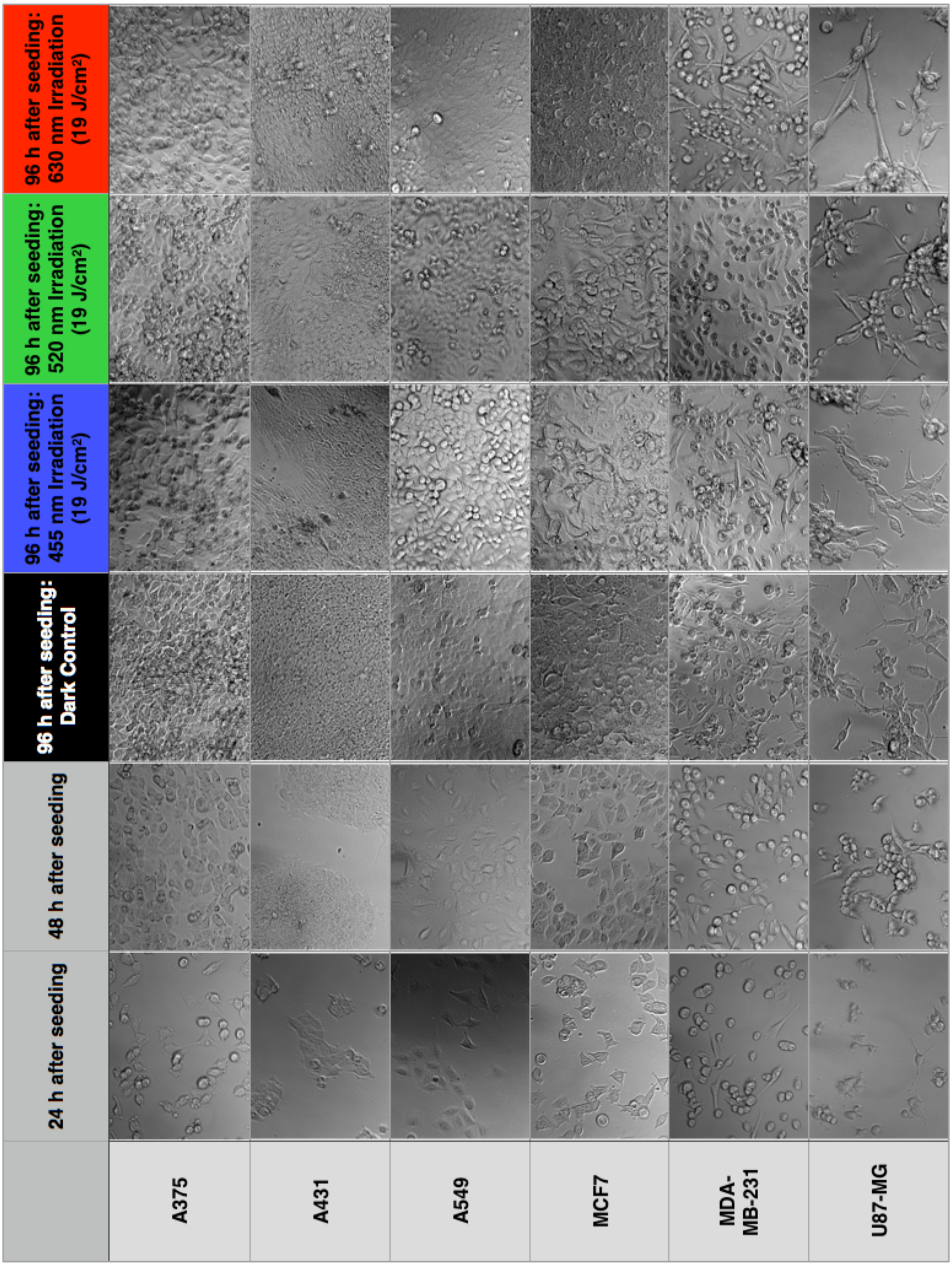

Figure S10. Micrographs (20X) of each cell line at 24 h, 48 h, and 96 h after seeding for dark populations and at 96 h for blue, green, or red irradiated cells (19 J·cm^{-2}).

VIII. Growth curve in the dark

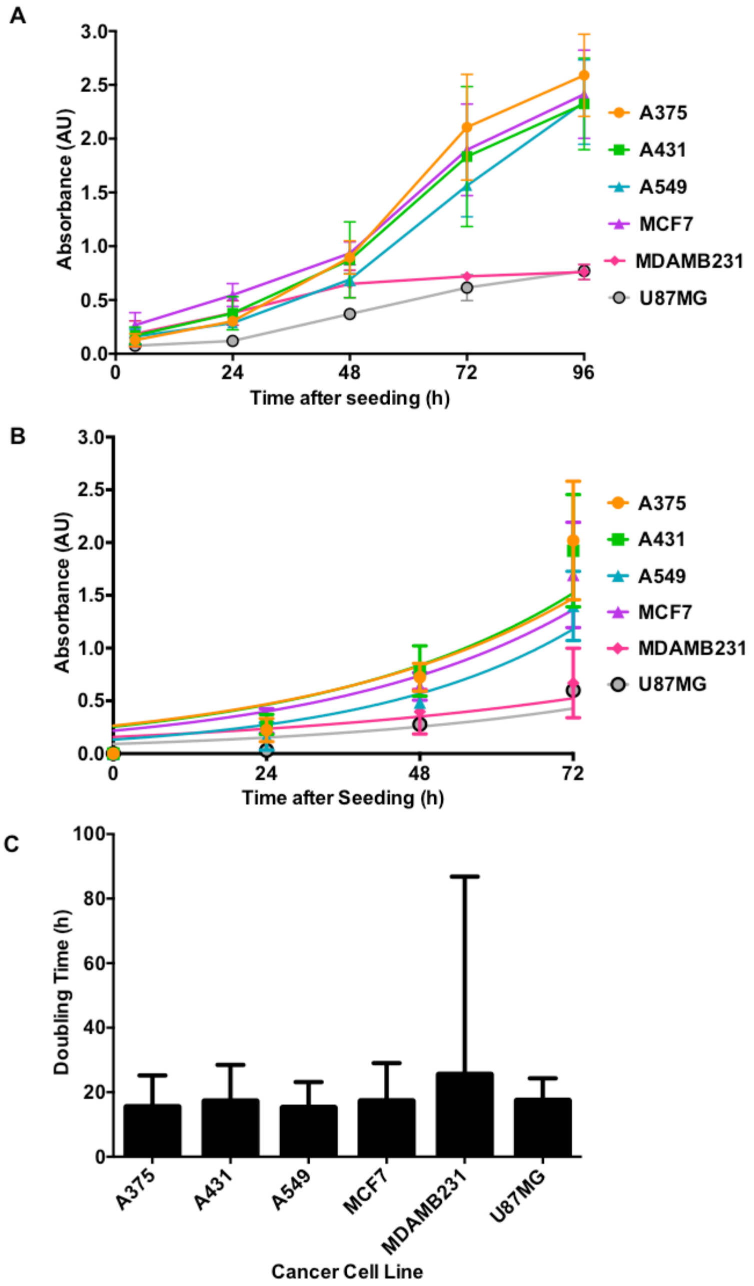

Figure S11. Growth curve (A), fitted exponential growth curves (B), and doubling times with 95% confidence interval (C) for A375 (orange), A431 (green), A549 (blue), MCF7 (purple), MDAMB231 (pink), and U-87 MG (gray) using SRB cell counting assay. Conditions: Cells were seeded at t = 0 at 7 x 10^3 (A375), 8 x 10^3 (A431), 5 x 10^3 (A549), 8 x 10^3 (MCF7), 1.2 x 10^4 (MDA-MB-231), and 6 x 10^3 (U-87 MG) cells/well in Opti-MEM complete without phenol red and incubated for 24 h at 37 °C and 7% CO_2. Opti-MEM complete without phenol red was added at 24 h and cells were incubated for an additional 24 h. Media was removed and refreshed and cells were placed back in the incubator. Cells were fixed using TCA at 4, 24, 48, 72, and 96 h after seeding and then stained with SRB. The SRB absorbance of ten technical replications was averaged for one experiment (n = 3). The absorbance values were exponentially fitted to determine the exponential growth curves using GraphPad Prism, non-linear fit of exponential growth. The doubling times were determined with 95% confidence intervals.

IX. Cancer cell line gene mutation information

Table S1. Cancer cell line with gene mutations and if available the protein sequence.[7]

Cancer cell	Blue light sensitive	Gene mutation name	Mutation protein sequence
**A-375**	Yes	BRAF<br>CDKN2A	p.V600E<br>p.E61* and p.E69*
**A-431**		TP53	p.R273H
**A549**	Yes	KRAS<br>CDK2NA (−/−)	p.G12S
**HCT 116**	Yes	KRAS<br>CDKN2A<br>PIK3CA	p.G13D<br>p.R24fs*20<br>p.H1047R
**HT-29**	Yes	BRAF<br>APC<br>PIK3CA<br>SMAD4<br>TP53	p.V600E<br>p.E853*<br>p.P449T<br>p.Q311*<br>p.R273H
**MCF7**		CDKN2A (−/−)<br>PIK3CA	 p.E545K
**MDA-MB-231**	Yes	BRAF<br>KRAS<br>CDKN2A (−/−)<br>TP53	p.G464V<br>p.G13D<br><br>p.R280K
**U-87 MG**		CDK2NA (−/−)<br>PTEN (−/−)	

References

1. H. J. Kuhn, S. E. Braslavsky and R. Schmidt, *Pure Appl. Chem.*, 1989, **61**, 187-210.
2. W. D. Bowman and J. N. Demas, *J Phys Chem*, 1976, **80**, 2434-2435.
3. C. G. Hatchard and C. A. Parker, *Proc. R. Soc. Lond. A*, 1956, **235**, 518-536.
4. J. Lee and H. H. Seliger, *J Chem Phys*, 1964, **40**, 519-523.
5. M. C. DeRosa and R. J. Crutchley, *Coord Chem Rev*, 2002, **233-234**, 351-371.
6. J. P. Tardivo, A. Del Giglio, C. S. de Oliveira, D. S. Gabrielli, H. C. Junqueira, D. B. Tada, D. Severino, R. de Fátima Turchiello and M. S. Baptista, *Photodiagnosis and Photodynamic Therapy*, 2005, **2**, 175-191.
7. ATCC, 2014, pp. 1-40.
